# Supplementary material for: Comparison of survival outcomes and anatomically specific severe injuries following traffic accidents among occupants of standard and K-car vehicles: A retrospective cohort study at a teaching hospital in Japan
Source: PLoS One. 2025 Feb 5;20(2):e0318748. doi: 10.1371/journal.pone.0318748 (PMC11798441; doi:10.1371/journal.pone.0318748)
Supplement: S1 Fig — Distribution of propensity scores in the unmatched (A) and matched groups (B). (PPTX) [file pone.0318748.s001.pptx]

## Slide 1
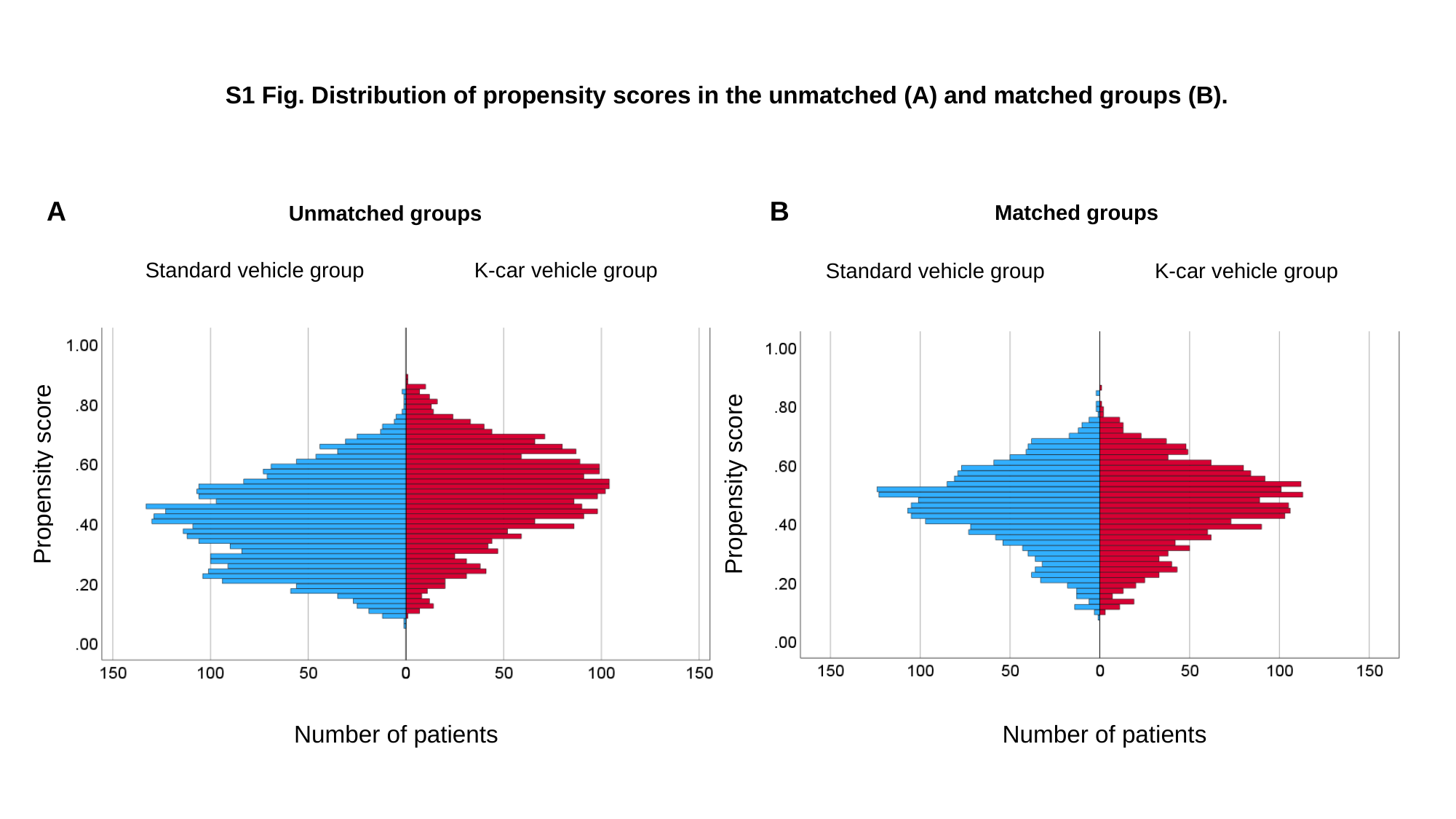

S1 Fig. Distribution of propensity scores in the unmatched (A) and matched groups (B).
B
A
Matched groups
Unmatched groups
Standard vehicle group
K-car vehicle group
Standard vehicle group
K-car vehicle group
Propensity score
Propensity score
Number of patients
Number of patients
