# Supplementary material for: Comparison of survival outcomes and anatomically specific severe injuries following traffic accidents among occupants of standard and K-car vehicles: A retrospective cohort study at a teaching hospital in Japan
Source: PLoS One. 2025 Feb 5;20(2):e0318748. doi: 10.1371/journal.pone.0318748 (PMC11798441; doi:10.1371/journal.pone.0318748)
Supplement: S2 Fig — The reference set was patients in the standard vehicle group. aPS adjustment, as described in the Methods. CI, confidence interval; OR, odds ratio; PS, propensity score. (PPTX) [file pone.0318748.s002.pptx]

## Slide 1
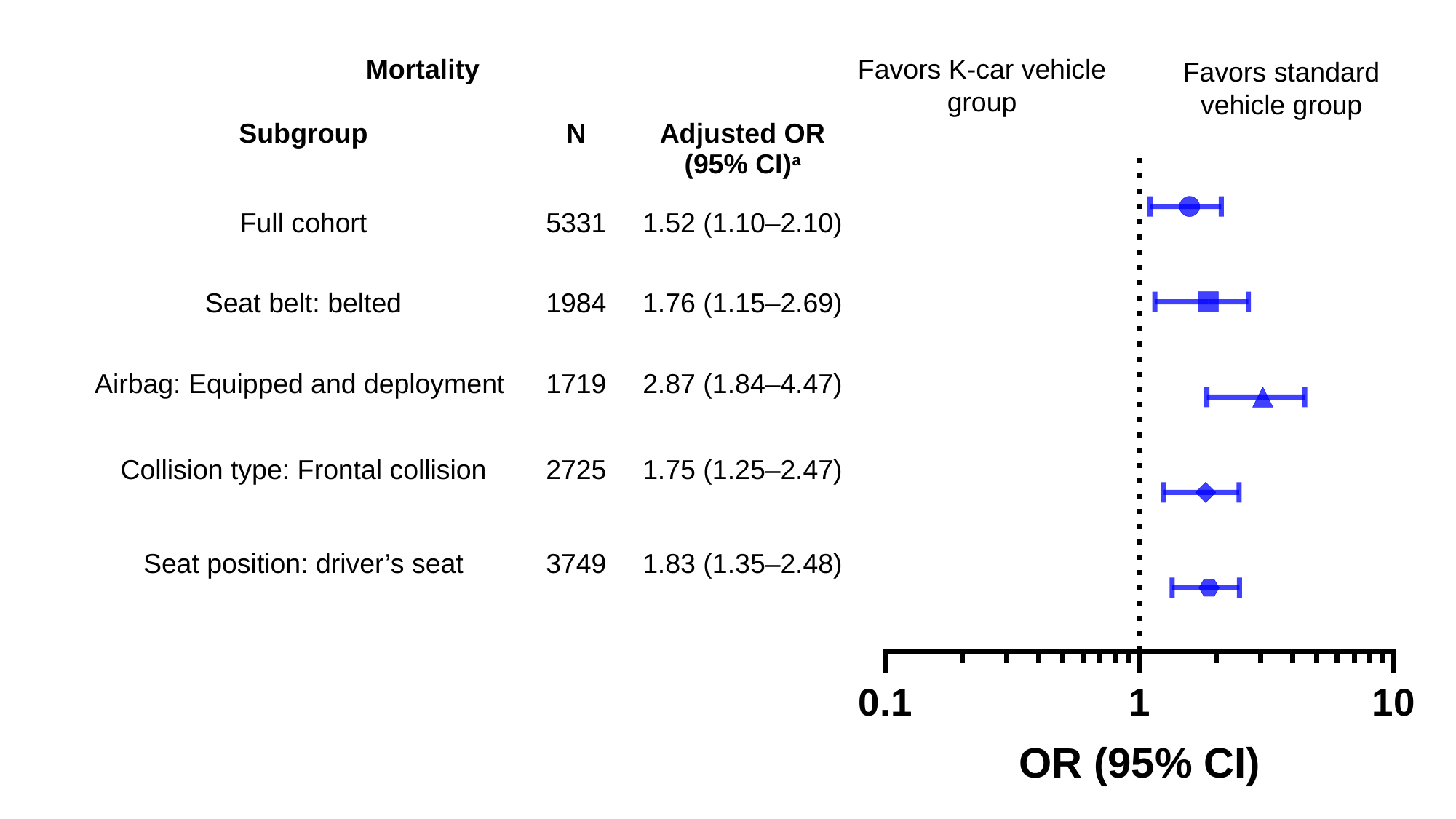

Mortality
Favors K-car vehicle group
Favors standard vehicle group
| Subgroup | N | Adjusted OR (95% CI)a |
| --- | --- | --- |
| Full cohort | 5331 | 1.52 (1.10–2.10) |
| Seat belt: belted | 1984 | 1.76 (1.15–2.69) |
| Airbag: Equipped and deployment | 1719 | 2.87 (1.84–4.47) |
| Collision type: Frontal collision | 2725 | 1.75 (1.25–2.47) |
| Seat position: driver’s seat | 3749 | 1.83 (1.35–2.48) |
