# Supplementary material for: Comparison of survival outcomes and anatomically specific severe injuries following traffic accidents among occupants of standard and K-car vehicles: A retrospective cohort study at a teaching hospital in Japan
Source: PLoS One. 2025 Feb 5;20(2):e0318748. doi: 10.1371/journal.pone.0318748 (PMC11798441; doi:10.1371/journal.pone.0318748)
Supplement: S3 Fig — The reference set was the standard vehicle group. aPS adjustment, as described in the Methods. CI, confidence interval; ISS, Injury Severity Score; OR, odds ratio; PS, propensity score. (PPTX) [file pone.0318748.s003.pptx]

## Slide 1
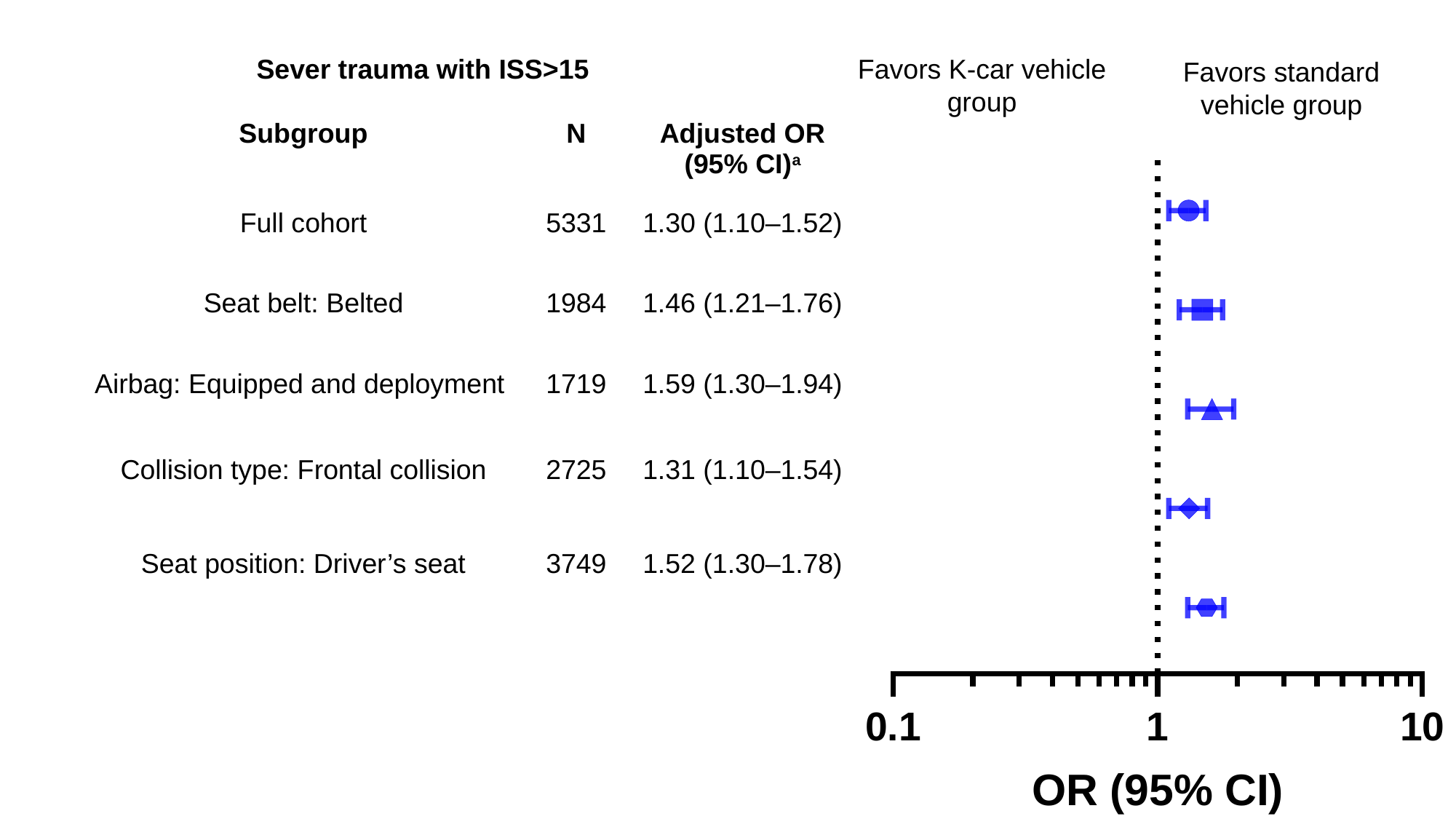

Sever trauma with ISS>15
Favors K-car vehicle group
Favors standard vehicle group
| Subgroup | N | Adjusted OR (95% CI)a |
| --- | --- | --- |
| Full cohort | 5331 | 1.30 (1.10–1.52) |
| Seat belt: Belted | 1984 | 1.46 (1.21–1.76) |
| Airbag: Equipped and deployment | 1719 | 1.59 (1.30–1.94) |
| Collision type: Frontal collision | 2725 | 1.31 (1.10–1.54) |
| Seat position: Driver’s seat | 3749 | 1.52 (1.30–1.78) |

## Slide 2
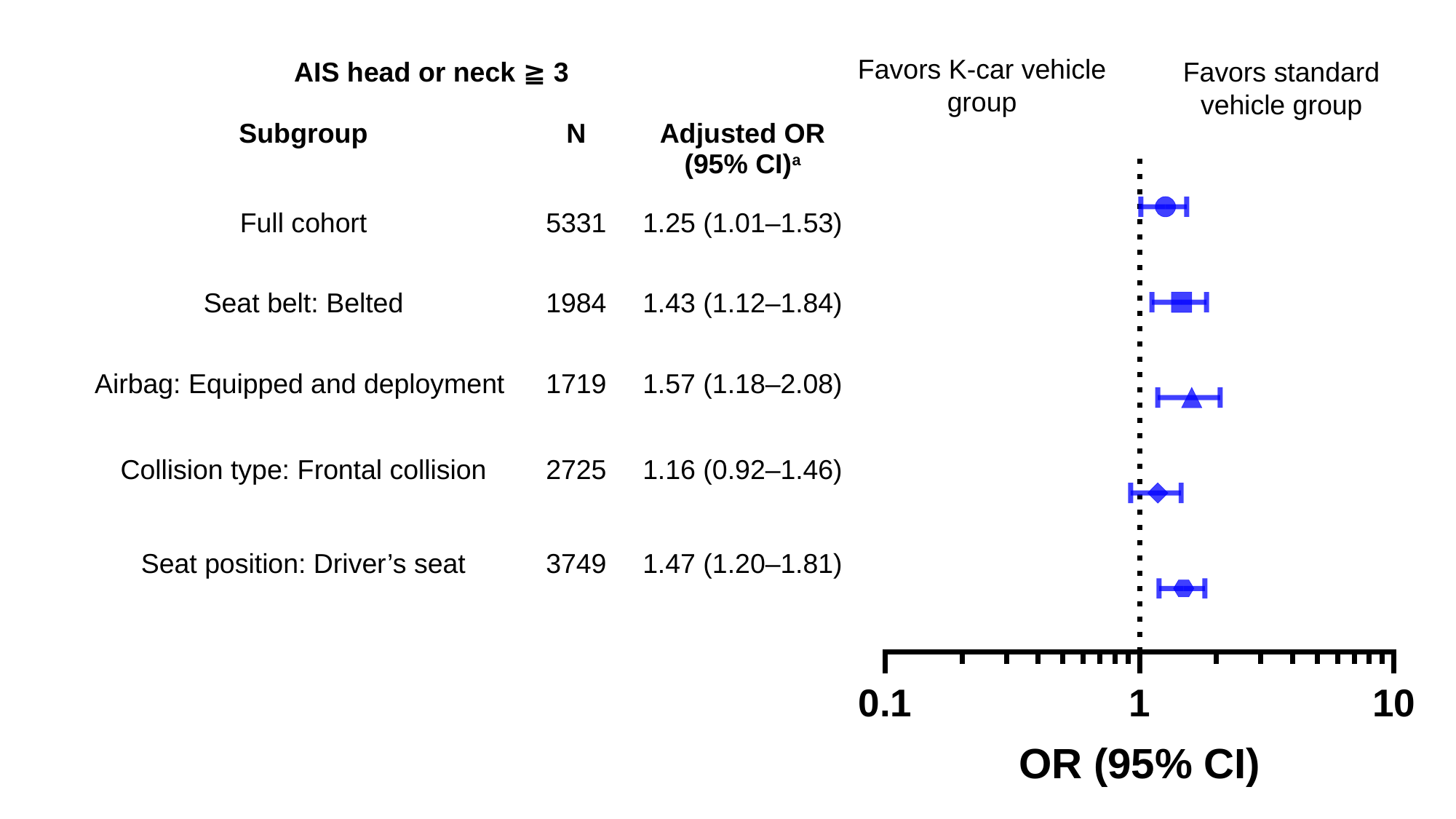

Favors K-car vehicle group
Favors standard vehicle group
AIS head or neck ≧ 3
| Subgroup | N | Adjusted OR (95% CI)a |
| --- | --- | --- |
| Full cohort | 5331 | 1.25 (1.01–1.53) |
| Seat belt: Belted | 1984 | 1.43 (1.12–1.84) |
| Airbag: Equipped and deployment | 1719 | 1.57 (1.18–2.08) |
| Collision type: Frontal collision | 2725 | 1.16 (0.92–1.46) |
| Seat position: Driver’s seat | 3749 | 1.47 (1.20–1.81) |

## Slide 3
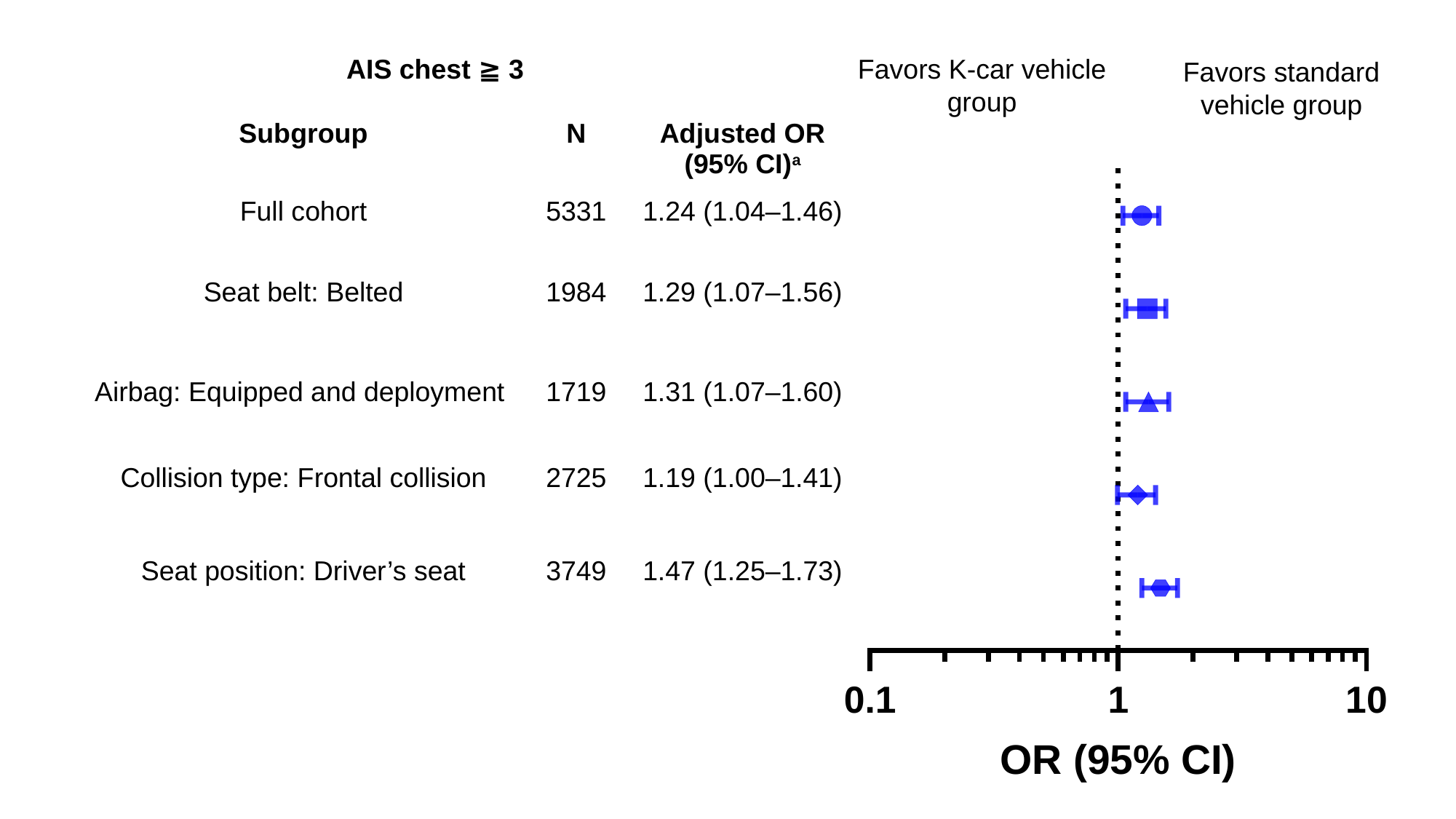

AIS chest ≧ 3
Favors K-car vehicle group
Favors standard vehicle group
| Subgroup | N | Adjusted OR (95% CI)a |
| --- | --- | --- |
| Full cohort | 5331 | 1.24 (1.04–1.46) |
| Seat belt: Belted | 1984 | 1.29 (1.07–1.56) |
| Airbag: Equipped and deployment | 1719 | 1.31 (1.07–1.60) |
| Collision type: Frontal collision | 2725 | 1.19 (1.00–1.41) |
| Seat position: Driver’s seat | 3749 | 1.47 (1.25–1.73) |

## Slide 4
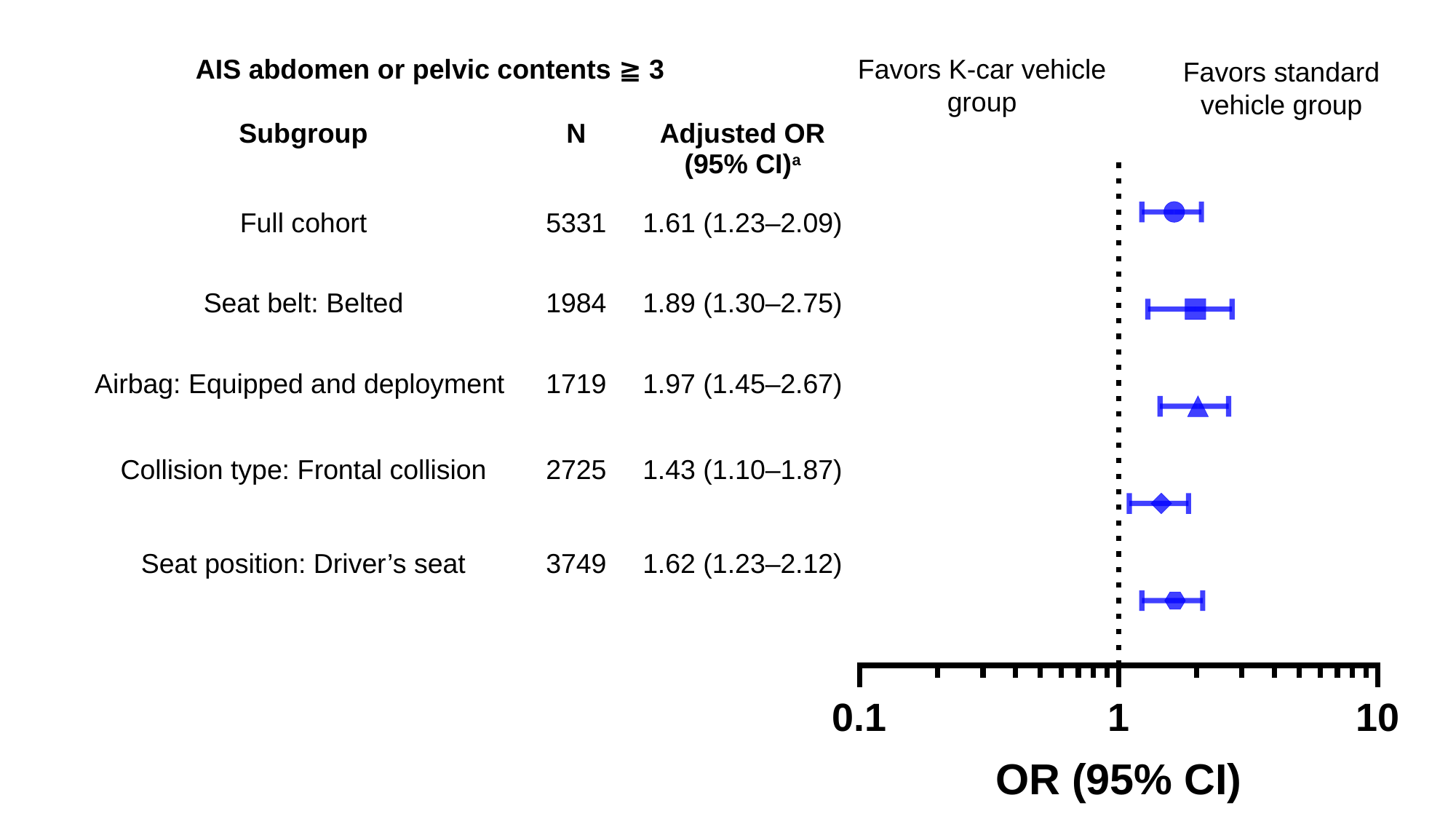

AIS abdomen or pelvic contents ≧ 3
Favors K-car vehicle group
Favors standard vehicle group
| Subgroup | N | Adjusted OR (95% CI)a |
| --- | --- | --- |
| Full cohort | 5331 | 1.61 (1.23–2.09) |
| Seat belt: Belted | 1984 | 1.89 (1.30–2.75) |
| Airbag: Equipped and deployment | 1719 | 1.97 (1.45–2.67) |
| Collision type: Frontal collision | 2725 | 1.43 (1.10–1.87) |
| Seat position: Driver’s seat | 3749 | 1.62 (1.23–2.12) |

## Slide 5
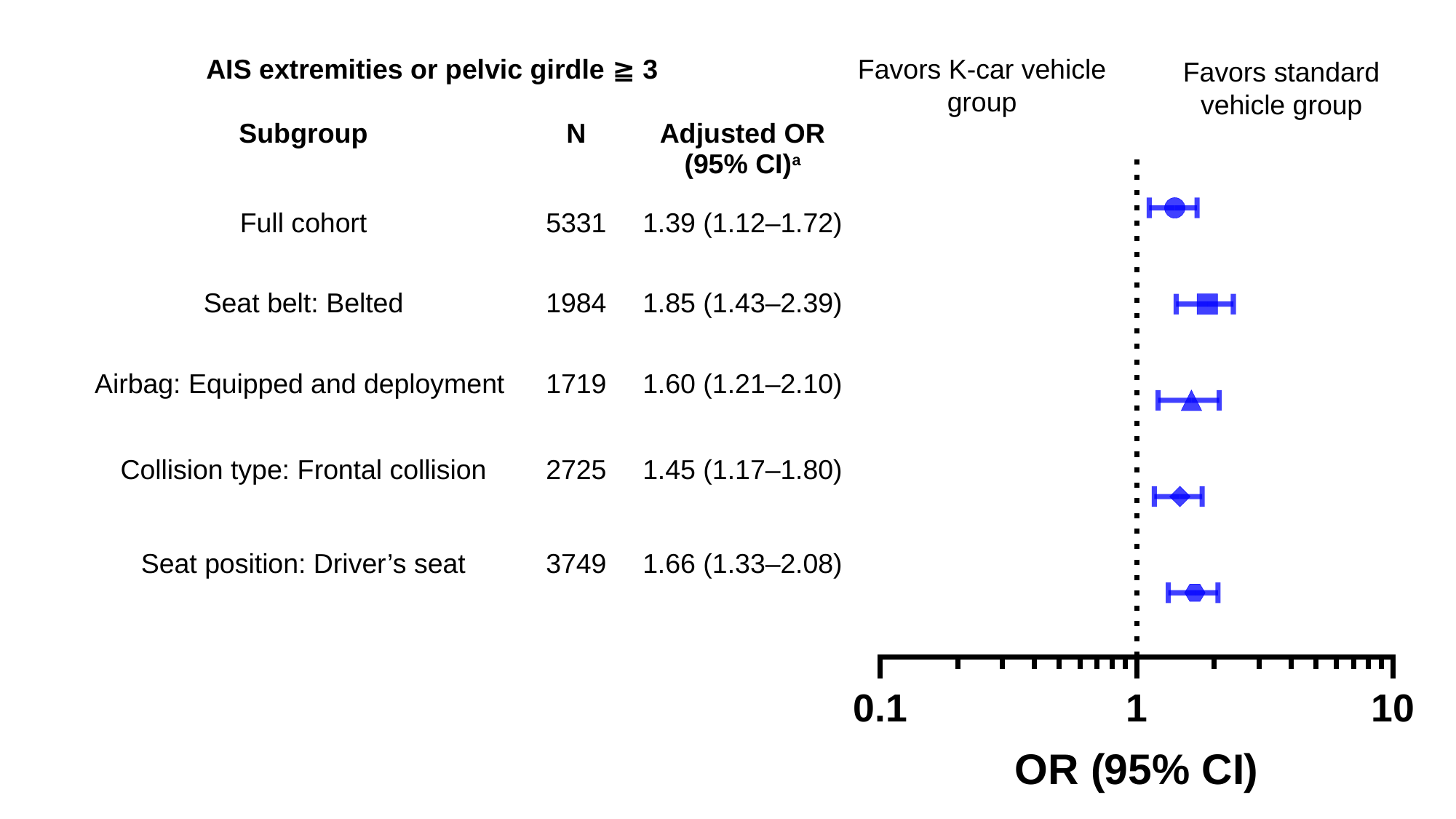

AIS extremities or pelvic girdle ≧ 3
Favors K-car vehicle group
Favors standard vehicle group
| Subgroup | N | Adjusted OR (95% CI)a |
| --- | --- | --- |
| Full cohort | 5331 | 1.39 (1.12–1.72) |
| Seat belt: Belted | 1984 | 1.85 (1.43–2.39) |
| Airbag: Equipped and deployment | 1719 | 1.60 (1.21–2.10) |
| Collision type: Frontal collision | 2725 | 1.45 (1.17–1.80) |
| Seat position: Driver’s seat | 3749 | 1.66 (1.33–2.08) |
