# Supplementary material for: Comparison of survival outcomes and anatomically specific severe injuries following traffic accidents among occupants of standard and K-car vehicles: A retrospective cohort study at a teaching hospital in Japan
Source: PLoS One. 2025 Feb 5;20(2):e0318748. doi: 10.1371/journal.pone.0318748 (PMC11798441; doi:10.1371/journal.pone.0318748)
Supplement: S4 Fig — (A, B) Prehospital LOS in the full (A) and PS-matched (B) cohort. (C, D) Hospital LOS in the full (C) and PS-matched (D) cohort. Prehospital LOS is defined as time from the emergency call to ED arrival. Hospital LOS is defined as time from hospital admission to hospital discharge or transfer. representing the data distribution (circles), mean (horizontal bar), and interquartile range (vertical bar), respectively. The p-values are derived from the Mann–Whitney U-test. LOS, length of stay; PS, propensity score; ED, emergency department. (PPTX) [file pone.0318748.s004.pptx]

## Slide 1
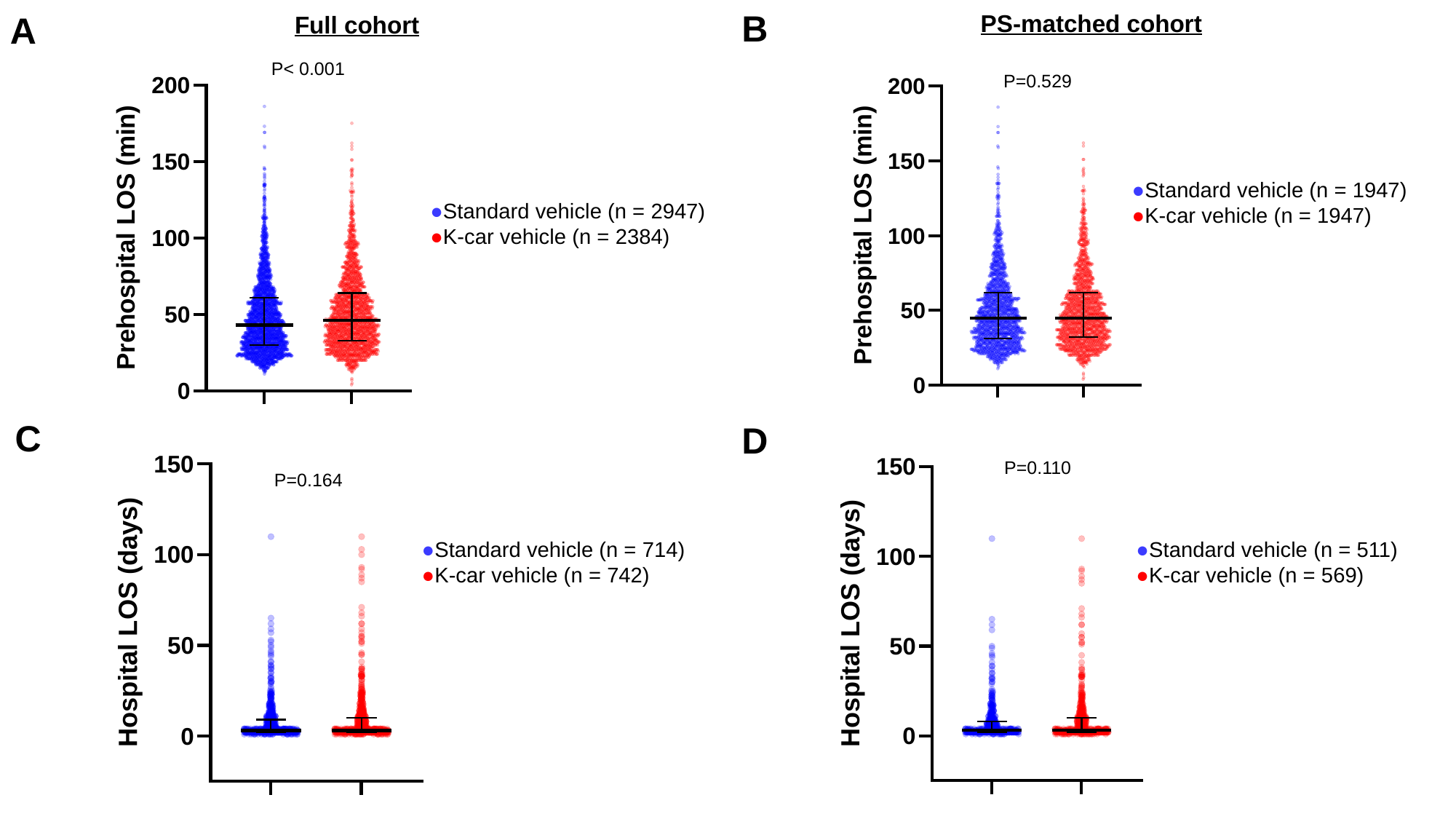

B
A
PS-matched cohort
Full cohort
P< 0.001
P=0.529
●Standard vehicle (n = 1947)
●K-car vehicle (n = 1947)
●Standard vehicle (n = 2947)
●K-car vehicle (n = 2384)
C
D
P=0.110
P=0.164
●Standard vehicle (n = 714)
●K-car vehicle (n = 742)
●Standard vehicle (n = 511)
●K-car vehicle (n = 569)
