# Supplementary material for: Comparison of survival outcomes and anatomically specific severe injuries following traffic accidents among occupants of standard and K-car vehicles: A retrospective cohort study at a teaching hospital in Japan
Source: PLoS One. 2025 Feb 5;20(2):e0318748. doi: 10.1371/journal.pone.0318748 (PMC11798441; doi:10.1371/journal.pone.0318748)
Supplement: S1 Table — (DOCX) [file pone.0318748.s005.docx]

# **S1 Table. Comparison of mortality rate:** **standard vehicle versus K-car vehicle.**

|  | **Full cohort** | | |  | **PS matched cohort** | | |
| --- | --- | --- | --- | --- | --- | --- | --- |
|  | **Standard vehicle (n=2947)** | **K-car vehicle (n=2384)** | **P** |  | **Standard vehicle (n=1947)** | **K-car vehicle (n=1947)** | **P** |
| **Outcome** |  |  | 0.004 |  |  |  | 0.019 |
| Dead | 77 (2.6) | 96 (4.0) |  |  | 51 (2.6) | 77 (4.0) |  |
| Alive | 2870 (97.4) | 2288 (96.0) |  |  | 1896 (97.4) | 1870 (96.0) |  |

Data are expressed as n (%). P values were derived using chi-squared tests.
