# Supplementary material for: Comparison of survival outcomes and anatomically specific severe injuries following traffic accidents among occupants of standard and K-car vehicles: A retrospective cohort study at a teaching hospital in Japan
Source: PLoS One. 2025 Feb 5;20(2):e0318748. doi: 10.1371/journal.pone.0318748 (PMC11798441; doi:10.1371/journal.pone.0318748)
Supplement: S2 Table — (DOCX) [file pone.0318748.s006.docx]

# **S2 Table. Comparison of anatomical injury severities:** **standard vehicle versus K-car vehicle.**

|  | **Full cohort** | | |  | **PS matched cohort** | | |
| --- | --- | --- | --- | --- | --- | --- | --- |
|  | **Standard vehicle (n=2947)** | **K-car vehicle (n=2384)** | **P** |  | **Standard vehicle (n=1947)** | **K-car vehicle (n=1947)** | **P** |
| **Severe trauma with ISS >15** |  |  | <0.001 |  |  |  | 0.003 |
| Yes | 366 (12.4) | 409 (17.2) |  |  | 243 (12.5) | 308 (15.8) |  |
| No | 2581 (87.6) | 1975 (82.8) |  |  | 1704 (87.5) | 1639 (84.2) |  |
| **AIS head or neck ≥3** |  |  | 0.030 |  |  |  | 0.125 |
| Yes | 221 (7.5) | 218 (9.1) |  |  | 143 (7.3) | 169 (8.7) |  |
| No | 2726 (92.5) | 2166 (90.9) |  |  | 1804 (92.7) | 1778 (91.3) |  |
| **AIS chest ≥3** |  |  | <0.001 |  |  |  | 0.010 |
| Yes | 333 (11.3) | 372 (15.6) |  |  | 222 (11.4) | 276 (14.2) |  |
| No | 2614 (88.7) | 2012 (84.4) |  |  | 1725 (88.6) | 1671 (85.8) |  |
| **AIS abdomen or pelvic contents ≥3** |  |  | <0.001 |  |  |  | 0.009 |
| Yes | 103 (3.5) | 131 (5.5) |  |  | 68 (3.5) | 101 (5.2) |  |
| No | 2844 (96.5) | 2253 (94.5) |  |  | 1879 (96.5) | 1846 (94.8) |  |
| **AIS extremities or pelvic girdle ≥3** |  |  | 0.003 |  |  |  | 0.015 |
| Yes | 171 (5.8) | 188 (7.9) |  |  | 112 (5.8) | 150 (7.1) |  |
| No | 2776 (94.2) | 2196 (92.1) |  |  | 1835 (94.2) | 1789 (91.9) |  |

Data are expressed as n (%). P values were derived using chi-squared tests. AIS, Abbreviated Injury Scale; ISS, Injury Severity Score.
