# Supplementary material for: Comparison of survival outcomes and anatomically specific severe injuries following traffic accidents among occupants of standard and K-car vehicles: A retrospective cohort study at a teaching hospital in Japan
Source: PLoS One. 2025 Feb 5;20(2):e0318748. doi: 10.1371/journal.pone.0318748 (PMC11798441; doi:10.1371/journal.pone.0318748)
Supplement: S3 Table — (DOCX) [file pone.0318748.s007.docx]

# **S3 Table Physiological severity among study participants.**

|  | **Full cohort** | | |  | **PS matched cohort** | | |
| --- | --- | --- | --- | --- | --- | --- | --- |
|  | **Standard vehicle**  **(n=2947)** | **K-car vehicle**  **(n=2384)** | **P** |  | **Standard vehicle**  **(n=1947)** | **K-car vehicle**  **(n=1947)** | **P** |
| **Physiological parameters** |  |  |  |  |  |  |  |
| **GCS score** |  |  | 0.097 |  |  |  | 0.083 |
| 13–15 | 2794 (94.8) | 2217 (93.0) |  |  | 1852 (95.1) | 1812 (93.1) |  |
| 9–12 | 41 (1.4) | 48 (2.0) |  |  | 28 (1.4) | 40 (2.1) |  |
| 6–8 | 28 (1.0) | 30 (1.3) |  |  | 12 (0.6) | 22 (1.1) |  |
| 4–5 | 13 (0.4) | 13 (0.5) |  |  | 10 (0.5) | 11 (0.6) |  |
| 3 | 71 (2.4) | 76 (3.2) |  |  | 45 (2.3) | 62 (3.2) |  |
| **Coma (GCS score <9)** |  |  | 0.034 |  |  |  | 0.025 |
| Yes | 112 (3.8) | 119 (5.0) |  |  | 67 (3.4) | 95 (4.9) |  |
| No | 2835 (96.2) | 2265 (95.0) |  |  | 1880 (96.6) | 1852 (95.1) |  |
| **SBP, mmHg** |  |  | 0.033 |  |  |  | 0.129 |
| >89 | 2854 (96.8) | 2272 (95.3) |  |  | 1882 (96.7) | 1857 (95.4) |  |
| 76–89 | 20 (0.7) | 23 (1.0) |  |  | 18 (0.9) | 16 (0.8) |  |
| 50–75 | 14 (0.5) | 25 (1.0) |  |  | 10 (0.5) | 21 (1.1) |  |
| 1–49 | 8 (0.3) | 7 (0.3) |  |  | 5 (0.3) | 6 (0.3) |  |
| 0 | 51 (1.7) | 57 (2.4) |  |  | 32 (1.6) | 47 (2.4) |  |
| **Shock (SBP <90 mmHg)** |  |  | 0.004 |  |  |  | 0.040 |
| Yes | 93 (3.2) | 112 (4.7) |  |  | 65 (3.3) | 90 (4.6) |  |
| No | 2854 (96.8) | 2272 (95.3) |  |  | 1882 (96.7) | 1857 (95.4) |  |
| **Respiratory rate, breaths/min** |  |  | 0.036 |  |  |  | 0.076 |
| >29 | 2793 (94.8) | 2211 (92.7) |  |  | 1846 (94.8) | 1808 (92.9) |  |
| 10–29 | 96 (3.3) | 111 (4.7) |  |  | 64 (3.3) | 89 (4.6) |  |
| 6–9 | 5 (0.2) | 3 (0.1) |  |  | 4 (0.2) | 3 (0.2) |  |
| 1–5 | 1 (0.03) | 1 (0.04) |  |  | 1 (0.1) | 0 (0.0) |  |
| 0 | 52 (1.8) | 58 (2.4) |  |  | 32 (1.6) | 47 (2.4) |  |

Data are expressed as n (%) unless otherwise noted.

P values were derived using chi-squared tests. GCS, Glasgow Coma Scale; PS, propensity score; SBP, systolic blood pressure.
