# Supplementary material for: Comparison of survival outcomes and anatomically specific severe injuries following traffic accidents among occupants of standard and K-car vehicles: A retrospective cohort study at a teaching hospital in Japan
Source: PLoS One. 2025 Feb 5;20(2):e0318748. doi: 10.1371/journal.pone.0318748 (PMC11798441; doi:10.1371/journal.pone.0318748)
Supplement: S4 Table — (DOCX) [file pone.0318748.s008.docx]

# **S4 Table. Requirement for emergency intervention among study participants.**

|  | **Full cohort** | | |  | **PS matched cohort** | | |
| --- | --- | --- | --- | --- | --- | --- | --- |
|  | **Standard vehicle**  **(n=2947)** | **K-car vehicle**  **(n=2384)** | **P** |  | **Standard vehicle**  **(n=1947)** | **K-car vehicle**  **(n=1947)** | **P** |
| **Emergency endotracheal intubation** |  |  | 0.023 |  |  |  | 0.046 |
| Yes | 139 (4.7) | 146 (6.1) |  |  | 90 (4.6) | 118 (6.1) |  |
| No | 2808 (95.3) | 2238 (93.9) |  |  | 1857 (95.4) | 1829 (93.9) |  |
| **Emergency surgery** |  |  | 0.001 |  |  |  | 0.001 |
| Yes | 229 (7.8) | 250 (10.5) |  |  | 145 (7.4) | 204 (10.5) |  |
| No | 2718 (92.2) | 2134 (89.5) |  |  | 1802 (92.6) | 1743 (89.5) |  |

Data are expressed as n (%) unless otherwise noted. P values were derived using chi-squared tests.
